# Supplementary material for: Cu- or Ag-containing Bi-Sb-Te for in-line roll-to-roll patterned thin-film thermoelectrics
Source: Nat Commun. 2025 Jan 3;16:196. doi: 10.1038/s41467-024-55279-7 (PMC11698979; doi:10.1038/s41467-024-55279-7)
Supplement: Supplementary file 1 — Supplementary Information [file 41467_2024_55279_MOESM1_ESM.pdf]

Cu- or Ag-containing Bi-Sb-Te for in-line roll-to-roll patterned thin-film thermoelectrics

Xudong Tao[1], Qianfang Zheng[1], Chongyang Zeng[2], Harry Potter[1], Zheng Zhang[1], Joshua Ellingford[3], Ruy S. Bonilla[1], Emiliano Bilotti[2], Patrick S. Grant[1], Hazel E. Assender[1]*

[1] Department of Materials, University of Oxford, Parks Road, Oxford, OX1 3PH, UK

[2] Department of Aeronautics, Imperial College London, Exhibition Road, London, SW7 2AZ, UK

[3] Plasma Quest Limited, Unit 1B, Rose Estate, Osborn Way, Hook, Hampshire RG27 9UT, UK

Corresponding Author: Xudong Tao, xt240@cam.ac.uk (Present address: Electrical Engineering Division, Department of Engineering, University of Cambridge, Cambridge, CB3 0FA, UK)

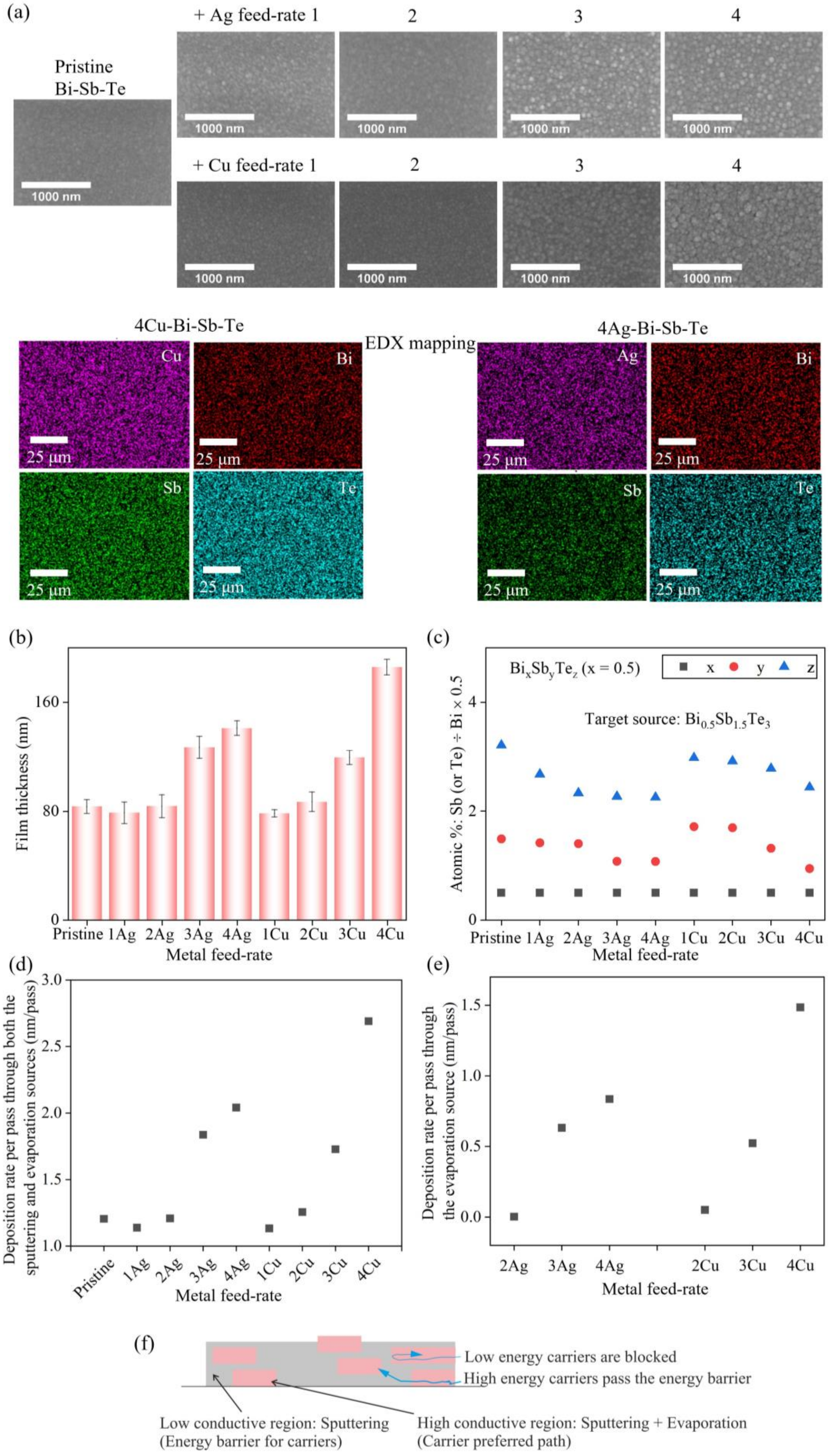
(a)
+ Ag feed-rate 1
2
3
4
Pristine
Bi-Sb-Te
1000 nm
+ Cu feed-rate 1
4Cu-Bi-Sb-Te
EDX mapping
4Ag-Bi-Sb-Te
Cu
Bi
Sb
Te
Ag
25 μm
(b)
Film thickness (nm)
Metal feed-rate
Pristine 1Ag 2Ag 3Ag 4Ag 1Cu 2Cu 3Cu 4Cu
(c)
$Bi_xSb_yTe_z$ (x = 0.5)
x y z
Target source: $Bi_{0.5}Sb_{1.5}Te_3$
Atomic %: Sb (or Te) ÷ Bi × 0.5
Metal feed-rate
(d)
Deposition rate per pass through both the sputtering and evaporation sources (nm/pass)
Metal feed-rate
(e)
Deposition rate per pass through the evaporation source (nm/pass)
2Ag 3Ag 4Ag 2Cu 3Cu 4Cu
Metal feed-rate
(f)
Low energy carriers are blocked
High energy carriers pass the energy barrier
Low conductive region: Sputtering
(Energy barrier for carriers)
High conductive region: Sputtering + Evaporation
(Carrier preferred path)

Figure S1. *(a) SEM and EDX images; (b) The film thickness (the error bar represents an average from ten different locations from the 1.8 m × 15 cm sample on the coating drum); (c) The corrected elemental composition (the atomic% of Bi is considered as a standard because there is only a slight change of Bi in Figure 2 c); (d & e) The deposition rate for each pass through the deposition sources; (f) Schematics of the heterogeneous structure featuring nano-scale, non-continuous layer-by-layer stacking.*

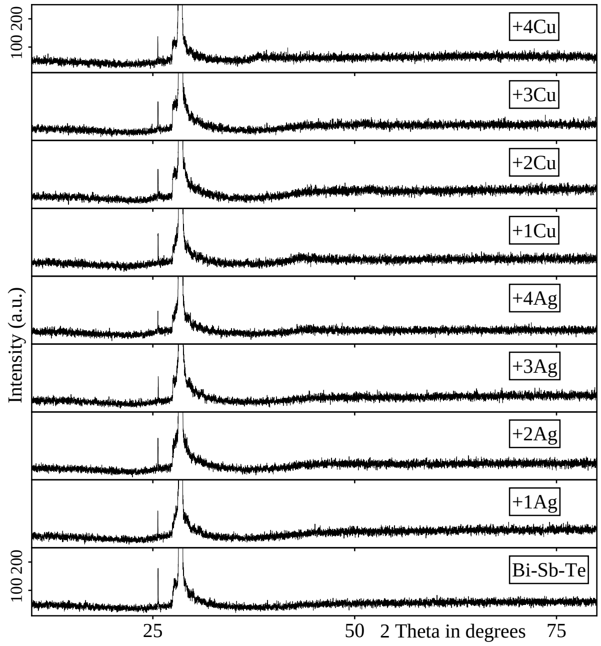

Figure S2. *XRD profiles of the films.*

Table S1. *Summary of p-type Bi-Sb-Te film (RT denotes room-temperature deposition).*

Reference	Substrate temperature [°C] and materials	Annealing [°C - h]	Thickness [nm]	Deposition technique	*PF* [$\times 10^{-4}$ W/(m K^2)]
[1], 2000	170 - Glass	200 - 1	315	Flash evaporation	16

[2], 2007	100 - SiO_2/Si	-	1000	Co-sputtering	1
		400 - 0.2			12
[3], 2008	200 - Glass	-	200	Flash evaporation	5
		300 - 0.5			34.9
[4], 2008	RT - Glass	300 - 16	850	Sputtering	20
[5], 2016	200 - Glass	-	500	Flash evaporation	5.4
		300 - 1			38.4
[6], 2020	400 - Polyimide	-	420	Co-sputtering	23.2
[7], 2020	350 - Glass	-	200	Sputtering	20
[8], 2020	300 - Sapphire	-	240	Co-sputtering	29
[9], 2020	RT - Glass	300 - 1	260	Thermal evaporation	0.1
[10], 2021	RT - SiO_2/Si	-	130	Ion beam sputtering	0.9
[11], 2021	100 - Glass	-	-	Co-sputtering	3.5
		300 - 1			10
[12], 2021	200 - SiO_2	-	1000	Sputtering	45.3
[13], 2022	100 -	-	100	Evaporation	1.3
		100 - 1			2.8
[14], 2023	RT - Polyimide	400 - 1	1500	Sputtering	36
[15], 2024	100 - Polyimide	300 - 1	-	Sputtering	11
[16], 2024	300 – Glass	300 – 0.5	800	Sputtering	2.2
This study	RT - PET	-	186	Sputtering +Evaporation	10.5

Table S2. *Comparison of room-temperature deposited Bi-Sb-Te film (referenced from Table S1).*

	Composition [atomic%]	Thickness [nm]	Resistivity [Ω cm]	Seebeck coefficient [μV/K]	*PF* [×10^{-4} W/(m K^2)]
[10], 2021	0.6 : 1.4 : 3	130	6 - 10	390 - 194	0.9
This work	0.47: 1.39: 3	83	1.3	113	0.01
	Cu-Bi-Sb-Te				10.5

Table S3. *Summary of Cu- or Ag-containing Bi-Sb-Te materials.*

Reference	Semiconductor type	Metal	Format	Hot processing	Fabrication technique	*PF* [×10^{-4} W/(m K^2)]
[17-21] , 2013 - 2023	p-type	Cu (< 2 at%)	Bulk	Yes	Sintering/Hot pressing/Solvent method	18 - 40
[22-24] , 2005 - 2021	p-type	Ag (< 2 at%)	Bulk	Yes	Sintering/Hot pressing	13 - 42
[25], 2019	p-type	Ag (1 at%)	Film	Yes	Printing	0.3
[26], 2022	p-type	Cu (0.3 at%)	Film	300°C - 1h anneal	Sputtering	8

[27], 2023	p-type	Ag (4 at%)	Film	300°C - 1h anneal	Sputtering	4
This study	p-type	Cu (68 at%)	Film	No	Sputtering + Evaporation	10.5
	n-type	Ag (14 at%)				1

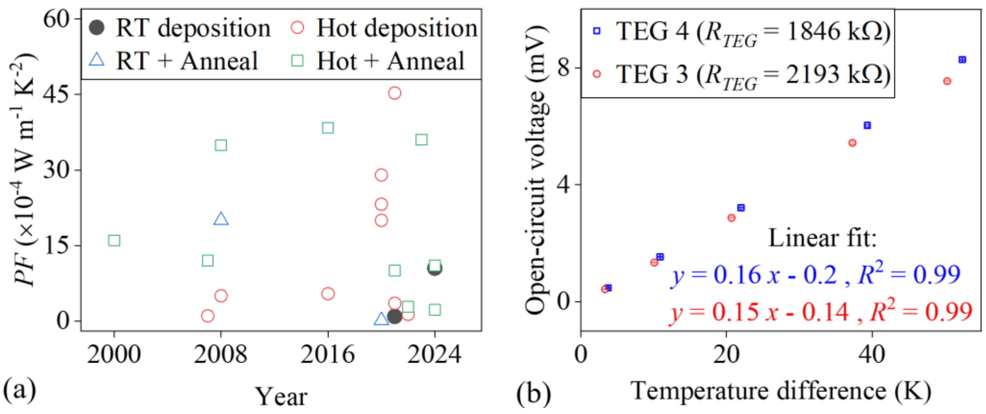

Figure S3. *(a) Summary of evaporated/sputtered Bi-Sb-Te materials from 2000 onwards (refer to Table S1 for detailed references); (b) Open-circuit voltage of devices 3 and 4, where the slope represents the Seebeck coefficient of the device.*

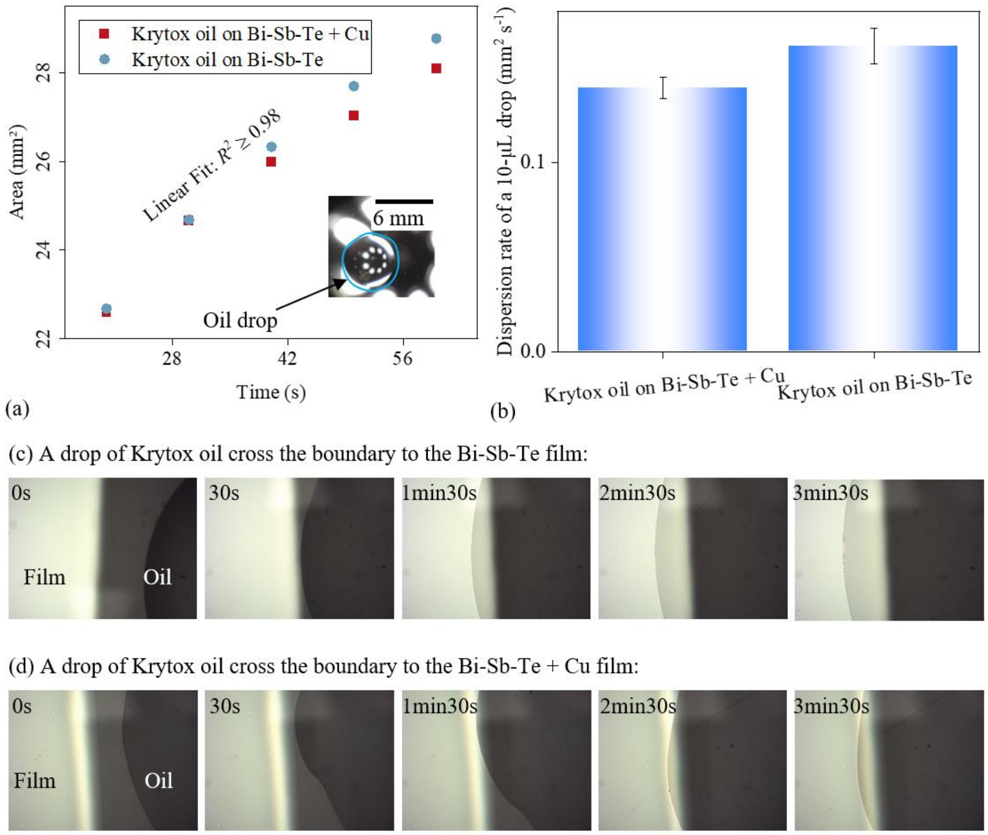

***Figure S4**. (a & b) The oil diffusion rate on the film surface of Bi-Sb-Te and Cu-Bi-Sb-Te (The error bar represents the mean of three measurements); (c & d) A drop of oil across the boundary of the substrate and the film.*

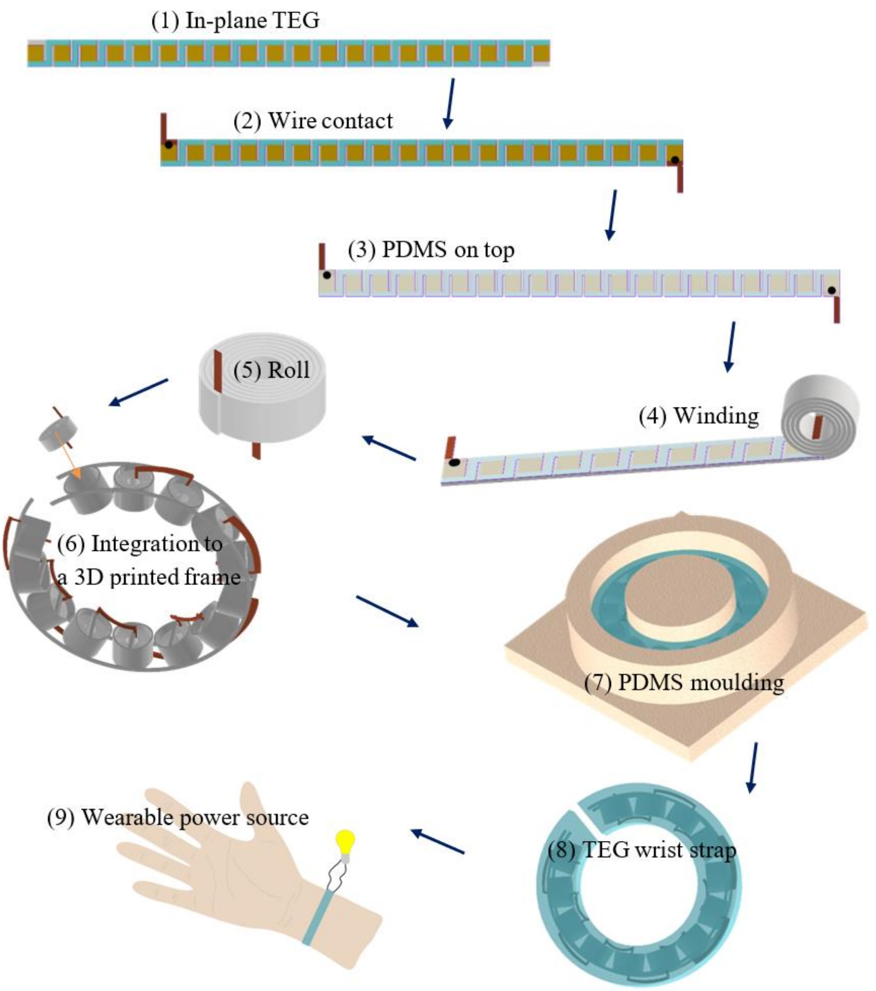

Figure S5. *Fabrication of wearable TEGs.*

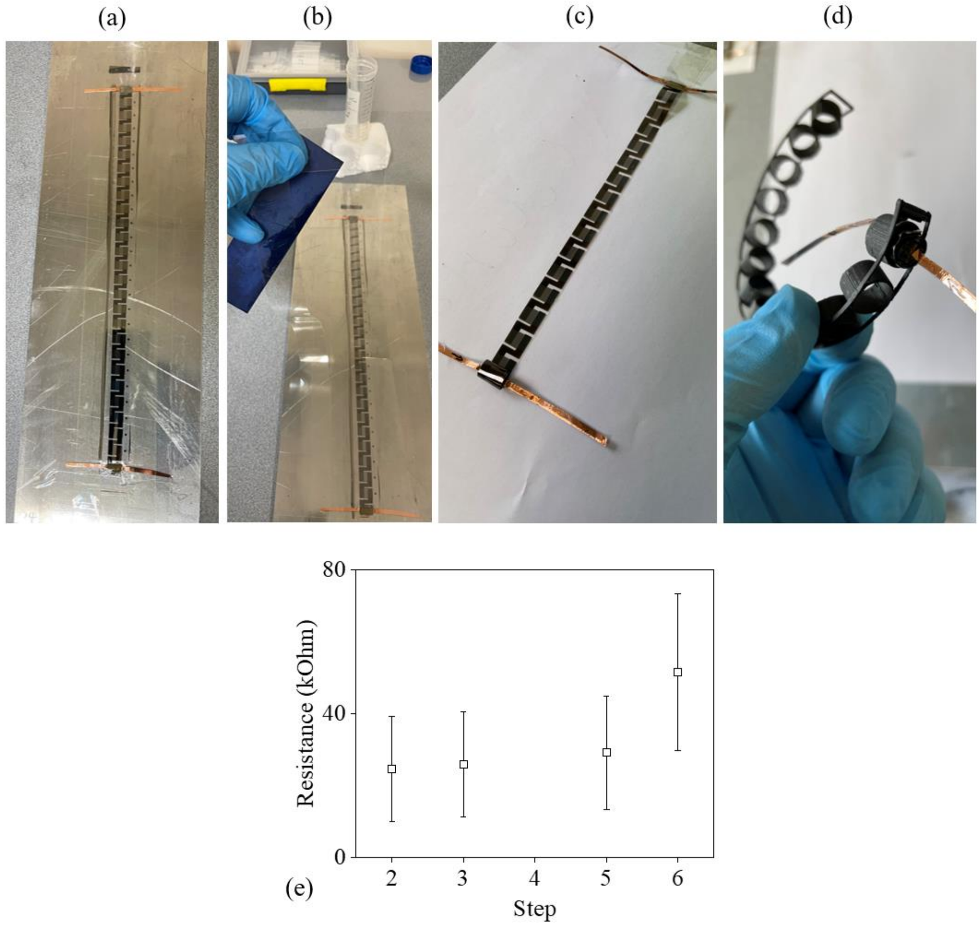

Figure S6. *Experimental steps illustrated in Figure S5: (a) Step 2 – Cu wire connection using Ag paste and cured in an oven; (b) Step 3 – Blade coating of PDMS on top, followed by oven curing; (c) Step 4 – Manual winding; (d) Step 6 – Integration into the frame; (e) Resistance change after each step, averaged from 20 samples (*The error bar represents the mean of three measurements*). The resistance result at Step 6 is from a single TEG cell in the frame, as shown in (d). After completing Step 8 in Figure S5, the resistance significantly increased to the MΩ range.*

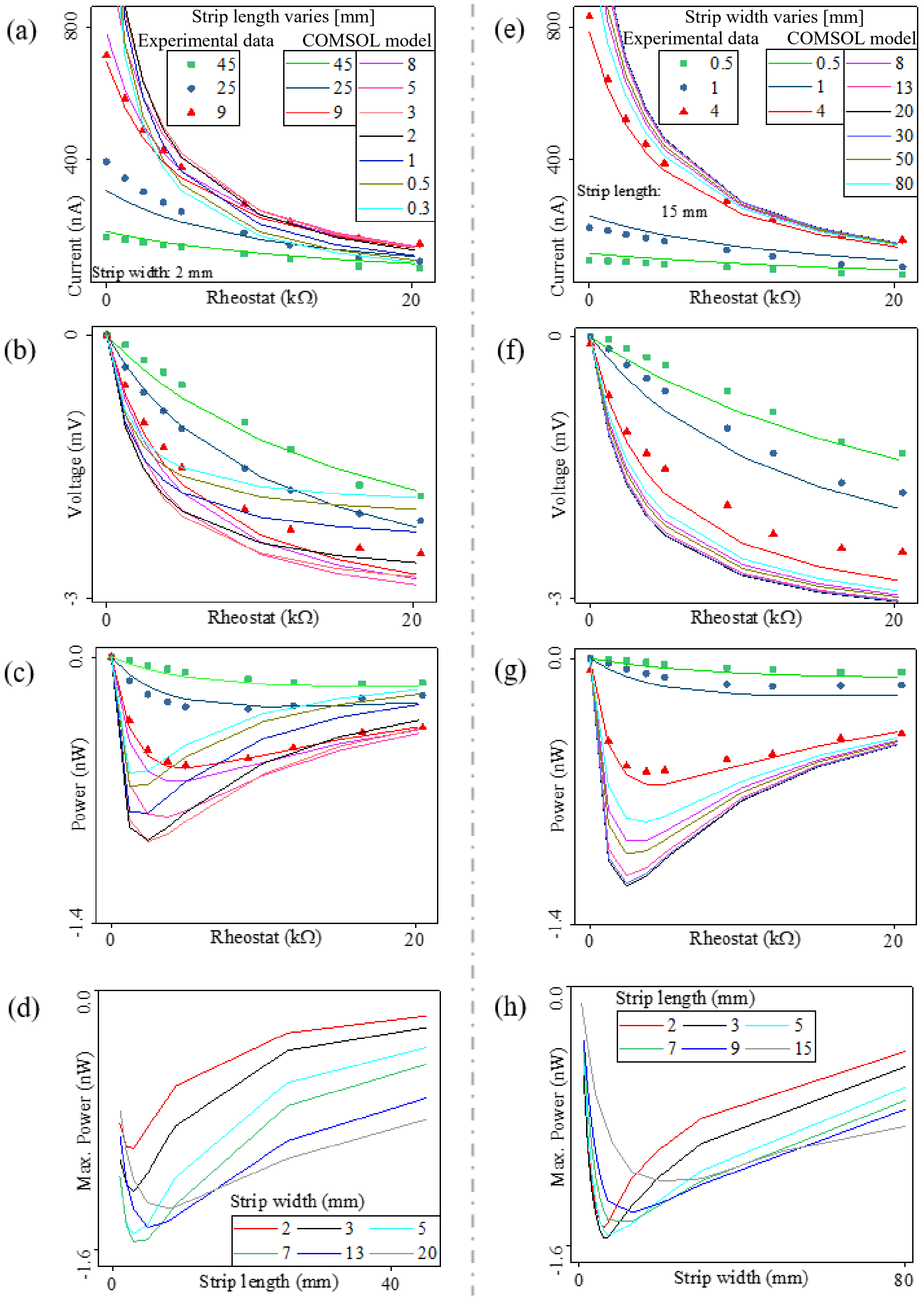

Figure S7. *The experimental data and the COMSOL simulated data of TEGs: (a-d) various lengths of the thermoelectric strip; (e-h) various widths of the thermoelectric strip. The strip length here represents the effective length (see Figure S8 a).*

COMSOL simulation analysis:

Our previous study [28] experimentally confirmed that a shorter and wider thermoelectric strip achieved a greater power output. The strip length was decreased to 13 mm and was not further decreased due to the practical constraints of the Seebeck measurement setup. However, it is evident that the strip length cannot be infinitely decreased since temperature difference (ΔT) will be affected, thus impacting the power output. In this study, the optimized strip dimension is predicted using COMSOL simulation (see **Figure S7**) and calibrated using the experimental data in [28]. As expected, there is an optimal length for thermoelectric strips of ~ 3 mm, at which the cold end (295 K) does not reach room temperature (293 K). This is attributed to a compromise between ΔT and strip thermal resistance: (1) A long strip can maintain the maximum ΔT, i.e., the maximum power output; (2) A long strip increases the resistance of the device, i.e., a decrease in current and thus power output; (3) Similarly, there is an optimal width for thermoelectric strips (6 mm), which should also be a compromise among several competitive mechanisms, as analyzed in [28]: (1) Intrinsic thermal resistances of a thermoelectric element; (2) Parasitic heat loss at interfacial resistance and contact electrodes; (3) The effect of sidewall radiative loss.

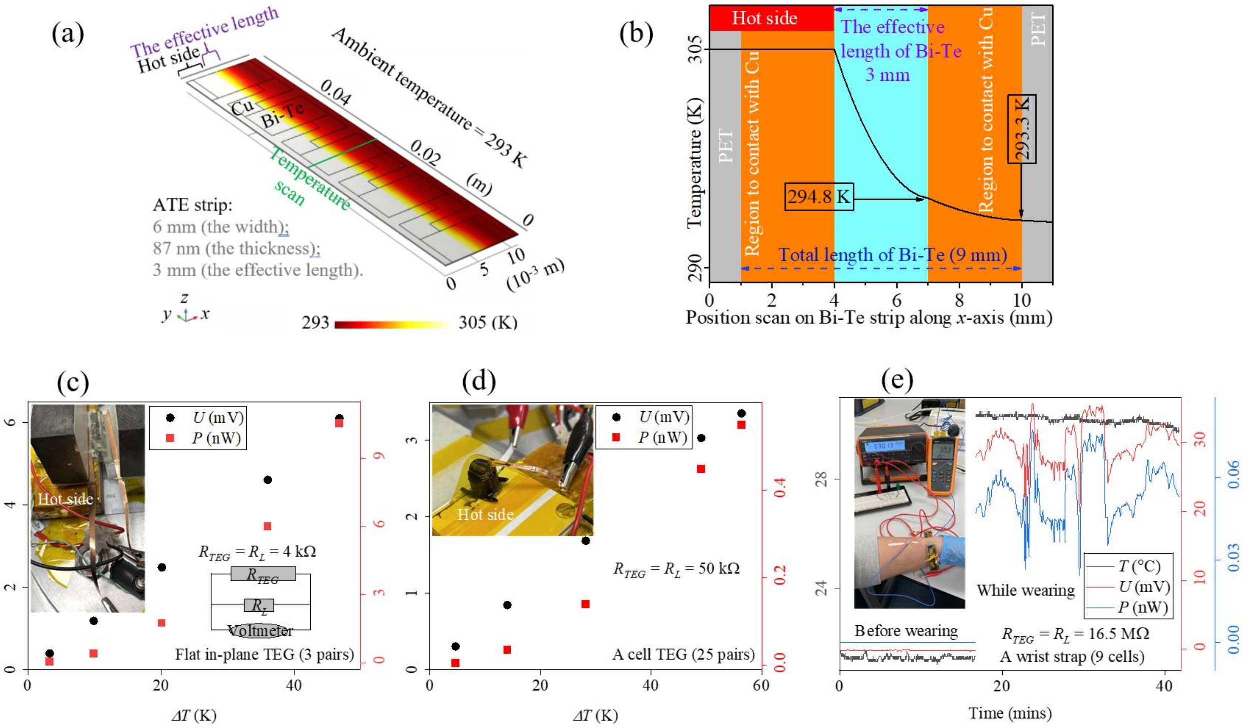

Figure S8. *(a) Heat transfer in a TEG; (b) Simulated temperature scan on top of a thermoelectric strip in (a). The effective length = the total length of the strip (9 mm) – the length of the contact at two ends (3 mm + 3mm). The thermoelectric power output of (c) a flat in-plane TEG with three thermoelectric pairs; (d) a cell TEG with 25 thermoelectric pairs; (e) a wrist strap with 9 cells.*

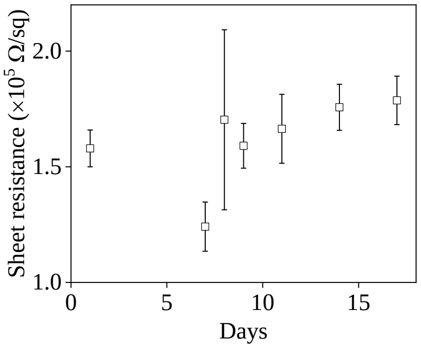

Figure S9*. Resistance variation of Bi-Sb-Te-based film over two weeks. The error bar represents the average of measurements taken from three identical samples.*

Reference

[1] Kim, I. , Electronic transport properties of the flash-evaporated p-type Bi0.5Sb1.5Te3 thermoelectric thin films, *Materials Letters.* 2000*, 44*, 75–79, https://doi.org/10.1016/S0167-577X(00)00005-7.

[2] Kim, D.; Lee, G.; Kim, O. , The influence of post-deposition annealing on thermoelectric properties of Bi–Sb–Te films prepared by sputtering, *Semiconductor Science and Technology.* 2007*, 22*, 132–136, https://doi.org/10.1088/0268-1242/22/2/023.

[3] TAKASHIRI, M.; MIYAZAKI, K.; TSUKAMOTO, H. , Structural and thermoelectric properties of fine-grained Bi0.4Te3.0Sb1.6 thin films with preferred orientation deposited by flash evaporation method, *Thin Solid Films.* 2008*, 516*, 6336–6343, https://doi.org/10.1016/j.tsf.2007.12.130.

[4] Bourgault, D.; Garampon, C. G.; Caillault, N.; Carbone, L.; Aymami, J. A. , Thermoelectric properties of n-type Bi2Te2.7Se0.3 and p-type Bi0.5Sb1.5Te3 thin films deposited by direct current magnetron sputtering, *Thin Solid Films.* 2008*, 516*, 8579–8583, https://doi.org/10.1016/j.tsf.2008.06.001.

[5] Takashiri, M.; Hamada, J. , Bismuth antimony telluride thin films with unique crystal orientation by two-step method, *Journal of Alloys and Compounds.* 2016*, 683*, 276–281, https://doi.org/10.1016/j.jallcom.2016.05.058.

[6] Hongjing Shang; Chaochao Dun; Yuan Deng; Taiguang Li; Zhaoshun Gao; Liye Xiao; Hongwei Gu; David J Singh; Zhifeng Ren; Fazhu Ding , Bi0.5Sb1.5Te3-based films for flexible thermoelectric devices, *Journal of Materials Chemistry. A, Materials for Energy and Sustainability.* 2020*, 8*, 4552–4561, https://doi.org/10.1039/C9TA13152C.

[7] Fan, P.; Li, R.; Chen, Y.; Zheng, Z.; Li, F.; Liang, G.; Luo, J. , High thermoelectric performance achieved in Bi0.4Sb1.6Te3 films with high (00l) orientation via magnetron sputtering, *Journal of the European Ceramic Society.* 2020*, 40*, 4016–4021, https://doi.org/10.1016/j.jeurceramsoc.2020.04.059.

[8] Han, X.; Zhang, Z.; Liu, Z.; Xu, C.; Lu, X.; Sun, L.; Jiang, P. , Effects of thickness on thermoelectric properties of Bi0.5Sb1.5Te3 thin films, *Applied Nanoscience.* 2020*, 10*, 2375–2381, https://doi.org/10.1007/s13204-020-01441-8.

[9] Marinho, A. A.; Costa, N. P.; Pereira, L. F. C.; Brito, F. A.; Chesman, C. , Thermoelectric properties of BiSbTe alloy nanofilms produced by DC sputtering: experiments and modeling, *Journal of Materials Science.* 2019*, 55*, 2429–2438, https://doi.org/10.1007/s10853-019-04188-y.

[10] Murmu, P. P.; Leveneur, J.; Storey, J. G.; Kennedy, J. , Effect of surface nanopatterning on the thermoelectric properties of bismuth antimony telluride films, *Materials Today : Proceedings*. 2021*, 36*, 416–420, https://doi.org/10.1016/j.matpr.2020.04.752.

[11] Liang, S.; Zhu, H.; Ge, X.; Yue, S. , Enhanced power factor of Bi0.5Sb1.5Te3 thin films via PbTe incorporating and annealing, *Surfaces and Interfaces*. 2021*, 24*, 101099, https://doi.org/10.1016/j.surfin.2021.101099.

[12] Tan, M.; Shi, X.; Liu, W.; Li, M.; Wang, Y.; Li, H.; Deng, Y.; Chen, Z. , Synergistic Texturing and Bi/Sb-Te Antisite Doping Secure High Thermoelectric Performance in Bi0.5Sb1.5Te3-Based Thin Films, *Advanced Energy Materials*. 2021*, 11*, n/a, https://doi.org/10.1002/aenm.202102578.

[13] Rad, D. Z.; Sajjadi, S. A.; Mansouri, H.; Saberi, Y. , The effect of annealing treatment on thermoelectric properties of nanostructured Bi0.5Sb1.5Te3 thin films fabricated by ball milling and thermal evaporation, *Journal of Nanoparticle Research : An Interdisciplinary Forum for Nanoscale Science and Technology*. 2022*, 24*, 264, https://doi.org/10.1007/s11051-022-05631-z.

[14] Qiu, G.; Li, J.; Ling, Y.; Dong, G.; Feng, J.; Zhang, P.; Liu, R. , Carrier concentration and orientation optimization for high performance (Sb,Bi)2Te3 thermoelectric films via magnetron co-sputtering, *Journal of Alloys and Compounds*. 2023*, 950*, 169916, https://doi.org/10.1016/j.jallcom.2023.169916.

[15] Hu, D.; Liang, S.; He, Y.; Zhang, R.; Yue, S. , Effects of working pressure during magnetron sputtering on thermoelectric performance of flexible p-type Bi0.5Sb1.5Te3 thin films, *Journal of Vacuum Science and Technology. B, Nanotechnology & Microelectronics*. 2024*, 42*, https://doi.org/10.1116/6.0003631.

[16] Zhang, R.; Jiang, Q.; Ye, H. Enhancement of the thermoelectric performance of (BiSb)2Te3 films by single target sputtering. *Ceramics international* **2024***, 50*, 24932–24938, DOI: 10.1016/j.ceramint.2024.02.167.

[17] Li, H.; Jing, H.; Han, Y.; Xu, Y.; Lu, G.; Xu, L. , Microstructure and transport properties of copper-doped p-type BiSbTe alloy prepared by mechanical alloying and subsequent spark plasma sintering, *Journal of Alloys and Compounds*. 2013*, 576*, 369–374, https://doi.org/10.1016/j.jallcom.2013.05.228.

[18] Lee, K. H.; Shin, W. H.; Kim, H.; Cho, H.; Kim, S. W.; Kim, S. , Important role of Cu in suppressing bipolar conduction in Bi-rich (Bi,Sb)2Te3, *Scripta Materialia*. 2020*, 186*, 225–229, https://doi.org/10.1016/j.scriptamat.2020.05.039.

[19] Lee, K.; Kim, H.; Kim, S.; Lee, E.; Lee, S.; Rhyee, J.; Jung, J.; Kim, I.; Wang, Y.; Koumoto, K. , Enhancement of Thermoelectric Figure of Merit for Bi0.5Sb1.5Te3 by Metal Nanoparticle Decoration, *Journal of Electronic Materials*. 2012*, 41*, 1165–1169, https://doi.org/10.1007/s11664-012-1913-0.

[20] Shi, F.; Wang, H.; Zhang, Q.; Tan, X.; Yin, Y.; Hu, H.; Li, Z.; Noudem, J. G.; Liu, G.; Jiang, J. , Improved Thermoelectric Properties of BiSbTe-AgBiSe2 Alloys by Suppressing Bipolar Excitation, *ACS Applied Energy Materials.* 2021*, 4*, 2944–2950, https://doi.org/10.1021/acsaem.1c00388.

[21] Liu, Y.; Tang, Y.; Tao, Y.; Zhang, Y.; Shen, L.; Ge, W.; Deng, S. , Ultralow thermal conductivity and high thermoelectric performance induced by multiscale lattice defects in Cu-doped BST alloys, *CrystEngComm.* 2023*, 26*, 1–19, https://doi.org/10.1039/D3CE00951C.

[22] Cui, J. L.; Xu, X. B. , Transport properties of quaternary Ag–Bi–Sb–Te alloys prepared by pressureless sintering, *Materials Letters.* 2005*, 59*, 3205–3208, https://doi.org/10.1016/j.matlet.2005.05.039.

[23] Lee, J. K.; Park, S.; Ryu, B.; Lee, H. S.; Park, J.; Park, S. , Effect of defect interactions with interstitial Ag in the lattice of BixSb2−xTe3 alloys and their thermoelectric properties, *Applied Physics Letters.* 2021*, 118*, https://doi.org/10.1063/5.0040808.

[24] Madavali, B.; Sharief, P.; Park, K.; Song, G.; Back, S.; Rhyee, J.; Hong, S. , Development of High-Performance Thermoelectric Materials by Microstructure Control of P-Type BiSbTe Based Alloys Fabricated by Water Atomization, *Materials.* 2021*, 14*, 4870, https://doi.org/10.3390%2Fma14174870.

[25] Cho, Y. M.; Gwon, G. H.; Kim, S. H.; Kim, D. W.; Choe, J.; Kim, K. T. , Fabrication of Silver-Doped (Bi,Sb)2Te3 Thermoelectric Film Prepared from Ag Nanoparticles/Bi-Sb-Te Pastes, *Journal of Nanoscience and Nanotechnology.* 2019*, 19*, 4270–4275, https://doi.org/10.1166/jnn.2019.16271.

[26] Liang, S.; zhang, M.; Zhu, H.; Yue, S. , Prominently Optimized Thermoelectric Performance Via Magnified Carrier Energy Filtering Effect in P-Type Cu-Doped Bi0.5sb1.5te3 Films, *SSRN Electronic Journal.* , https://dx.doi.org/10.2139/ssrn.4211235.

[27] Liang, S.; Shi, Y.; Hu, D.; Zhu, H.; Yue, S. , Significantly improved thermoelectric performance of Ag-doped Bi0.5Sb1.5Te3 films deriving from high-efficient carrier filtering effect, *Ceramics International.* 2023*, 49*, 35309–35315, https://doi.org/10.1016/j.ceramint.2023.08.203.

[28] Tao, X.; Zhang, K.; Gregory, D.; Liu, J.; Assender, H. E. , Device Optimization and Large-Scale Roll-to-Roll Manufacturability of Flexible Thin-Film Thermoelectric Generators, *Energy Technology (Weinheim, Germany).* 2021*, 9*, n/a, https://doi.org/10.1002/ente.202001008.
