# Supplementary material for: Cu- or Ag-containing Bi-Sb-Te for in-line roll-to-roll patterned thin-film thermoelectrics
Source: Nat Commun. 2025 Jan 3;16:196. doi: 10.1038/s41467-024-55279-7 (PMC11698979; doi:10.1038/s41467-024-55279-7)
Supplement: Supplementary file 2 — Description of Additional Supplementary Files [file 41467_2024_55279_MOESM2_ESM.pdf]

Description of Additional Supplementary Files

Supplementary Movie 1: Oxford Selective Metallisation Technique in Roll-to-Roll
