# Supplementary material for: Cu- or Ag-containing Bi-Sb-Te for in-line roll-to-roll patterned thin-film thermoelectrics
Source: Nat Commun. 2025 Jan 3;16:196. doi: 10.1038/s41467-024-55279-7 (PMC11698979; doi:10.1038/s41467-024-55279-7)
Supplement: Supplementary file 4 — Reporting Summary [file 41467_2024_55279_MOESM4_ESM.pdf]

Corresponding author(s): Xudong Tao

Lasing Reporting Summary

Nature Research wishes to improve the reproducibility of the work that we publish. This form is intended for publication with all accepted papers reporting claims of lasing and provides structure for consistency and transparency in reporting. Some list items might not apply to an individual manuscript, but all fields must be completed for clarity.

For further information on Nature Research policies, including our data availability policy, see Authors & Referees.

• Experimental design

Please check: are the following details reported in the manuscript?

1. Threshold

Item	Yes/No	Comment
Plots of device output power versus pump power over a wide range of values indicating a clear threshold	☐ Yes ☒ No	No lasers were used in our study

2. Linewidth narrowing

Item	Yes/No	Comment
Plots of spectral power density for the emission at pump powers below, around, and above the lasing threshold, indicating a clear linewidth narrowing at threshold	☐ Yes ☒ No	No lasers were used in our study
Resolution of the spectrometer used to make spectral measurements	☐ Yes ☒ No	No lasers were used in our study

3. Coherent emission

Item	Yes/No	Comment
Measurements of the coherence and/or polarization of the emission	☐ Yes ☒ No	No lasers were used in our study

4. Beam spatial profile

Item	Yes/No	Comment
Image and/or measurement of the spatial shape and profile of the emission, showing a well-defined beam above threshold	☐ Yes ☒ No	No lasers were used in our study

5. Operating conditions

Item	Yes/No	Comment
Description of the laser and pumping conditions<br>*Continuous-wave, pulsed, temperature of operation*	☐ Yes ☒ No	No lasers were used in our study
Threshold values provided as density values (e.g. W cm^{-2} or J cm^{-2}) taking into account the area of the device	☐ Yes ☒ No	No lasers were used in our study

6. Alternative explanations

Item	Yes/No	Comment
Reasoning as to why alternative explanations have been ruled out as responsible for the emission characteristics<br>*e.g. amplified spontaneous, directional scattering; modification of fluorescence spectrum by the cavity*	☐ Yes ☒ No	No lasers were used in our study

7. Theoretical analysis

Item	Yes/No	Comment
Theoretical analysis that ensures that the experimental values measured are realistic and reasonable<br>*e.g. laser threshold, linewidth, cavity gain-loss, efficiency*	☐ Yes ☒ No	No lasers were used in our study

8. Statistics

Item	Yes/No	Comment
Number of devices fabricated and tested	☐ Yes ☒ No	No lasers were used in our study
Statistical analysis of the device performance and lifetime (time to failure)	☐ Yes ☒ No	No lasers were used in our study
